# The Role of Bovine Kappa-Casein Glycomacropeptide in Modulating the Microbiome and Inflammatory Responses of Irritable Bowel Syndrome

**DOI:** 10.3390/nu15183991

**Published:** 2023-09-15

**Authors:** Yunyao Qu, Si Hong Park, David C. Dallas

**Affiliations:** 1 Department of Food Science & Technology, Oregon State University, Corvallis, OR 97331, USA; yunyao.qu@oregonstate.edu (Y.Q.); sihong.park@oregonstate.edu (S.H.P.); 2 Nutrition Program, College of Health, Oregon State University, Corvallis, OR 97331, USA

**Keywords:** irritable bowel syndrome (IBS), kappa-casein glycomacropeptide (GMP), gut microbiome, gut motility, gut barrier function, inflammation, therapeutic benefits

## Abstract

Irritable bowel syndrome (IBS) is a common gastrointestinal disorder marked by chronic abdominal pain, bloating, and irregular bowel habits. Effective treatments are still actively sought. Kappa-casein glycomacropeptide (GMP), a milk-derived peptide, holds promise because it can modulate the gut microbiome, immune responses, gut motility, and barrier functions, as well as binding toxins. These properties align with the recognized pathophysiological aspects of IBS, including gut microbiota imbalances, immune system dysregulation, and altered gut barrier functions. This review delves into GMP’s role in regulating the gut microbiome, accentuating its influence on bacterial populations and its potential to promote beneficial bacteria while inhibiting pathogenic varieties. It further investigates the gut microbial shifts observed in IBS patients and contemplates GMP’s potential for restoring microbial equilibrium and overall gut health. The anti-inflammatory attributes of GMP, especially its impact on vital inflammatory markers and capacity to temper the low-grade inflammation present in IBS are also discussed. In addition, this review delves into current research on GMP’s effects on gut motility and barrier integrity and examines the changes in gut motility and barrier function observed in IBS sufferers. The overarching goal is to assess the potential clinical utility of GMP in IBS management.

## 1. Introduction

### 1.1. Background on Irritable Bowel Syndrome

Irritable bowel syndrome (IBS) is a complex, chronic functional bowel disorder characterized by altered bowel habits and abdominal discomfort or pain [1]. Common symptoms include abdominal pain or discomfort, bloating, gas, diarrhea, and constipation; the disorder can severely impact an individual’s quality of life [2]. In some cases, IBS can also cause nausea, fatigue, and changes in appetite [3]. IBS is diagnosed based on a combination of symptoms and no single test can definitively diagnose the disorder.

Affecting 10–15% of the population in North America, 6–19% in Europe, and 3–20% in Asia [3,4,5], IBS has significantly impacted healthcare utilization and costs [6]. IBS has a higher prevalence among individuals assigned female at birth compared to males, with a female-to-male ratio of approximately 2:1 [7].

The exact cause of IBS remains unknown. However, it is thought to result from a combination of genetic, psychological, and environmental factors affecting the functioning of the digestive system [3,8].

### 1.2. Current Treatment Modalities for IBS

Current treatment strategies for IBS primarily focus on alleviating IBS symptoms [9]. Pharmacological approaches are varied, including the use of bulking agents, which have shown mixed results in alleviating constipation [10,11]. Antidiarrheal agents such as loperamide are effective at modulating stool consistency, though they do not address abdominal pain [12,13]. Antispasmodics aim to alleviate IBS pain by inhibiting muscle wall contractile pathways. However, their effectiveness remains unclear due to inconsistent clinical trial results and potential side effects such as exacerbating constipation [14,15]. The use of prokinetics, agents known to stimulate gastrointestinal motility, has been largely discouraged in treating IBS with constipation due to their proven ineffectiveness and associated cardiac toxicity, with some even being withdrawn from several countries [16,17,18]. Low doses of antidepressants have demonstrated potential for alleviating chronic abdominal pain, a prevalent symptom in IBS patients, by influencing the brain–gut axis [19,19,20]. Serotoninergic agents, such as cilansetron, are currently undergoing trials for their potential to reduce abdominal pain in IBS characterized by diarrhea (IBS-D), albeit with concerns about potential ischemic colitis [21]. Neurotrophins are also being examined for their ability to accelerate intestinal transit, though their precise role and safety profile in IBS management remains unclear [22]. Additionally, exploring tachykinin receptor antagonists is in its infancy, with preliminary studies indicating potential for alleviating various IBS symptoms. However, comprehensive clinical trials are needed to ascertain their efficacy and safety [23,24,25]. Somatostatin analogs may offer potential benefits in managing pain and severe diarrhea in IBS patients by modulating various brain centers involved in pain perception. However, their clinical application is hindered by the lack of practical administration methods and comprehensive clinical trials [26]. Adrenergic modulators are also under investigation, with some potentially improving abdominal discomfort and stool consistency. However, further studies are essential to determine their safety, especially considering the severe cardiac toxicity associated with some agents in this category [27,28].

Non-pharmacological interventions have also been applied to IBS management. For example, elimination diets can identify foods associated with symptoms for future avoidance [29]. The use of probiotics in IBS management is also being explored, but clinical evidence remains inconclusive [30]. Psychotherapy can address psychological factors intricately linked to IBS; for instance, stress management and cognitive behavioral therapy have shown promise for alleviating IBS symptoms [31].

Herein, we highlight the role of the gut microbiome and inflammation in the pathogenesis of IBS, and the potential to therapeutically target these underlying factors.

### 1.3. Importance of the Gut Microbiome and Inflammation in IBS Pathogenesis

Recent research has recognized the importance of the gut microbiome and low-grade inflammation in the pathogenesis of IBS [32,33,34]. Dysbiosis, or an imbalance in the gut microbiota composition, has been observed in IBS patients and may contribute to the onset and maintenance of IBS symptoms [35]. Studies indicate that ~73% of people with IBS have dysbiosis compared with 16% of otherwise healthy individuals [36,37,38,39]. Altered gut microbiota can lead to increased gut permeability, allowing for the translocation of bacterial components such as lipopolysaccharide (LPS) and the triggering of immune activation [40]. This immune activation can lead to low-grade chronic inflammation [32], which may further aggravate IBS symptoms. Thus, targeting the gut microbiota and inflammation has emerged as a promising therapeutic strategy for IBS [41].

### 1.4. Bovine Kappa-Casein Glycomacropeptide: A Potential Nutritional Intervention in IBS Management

Since IBS pathogenesis has been linked to altered gut microbiota composition and increased inflammation in the gut, nutritional interventions could potentially alleviate and positively influence symptoms. One nutritional supplement that can potentially improve the dysregulated physiology in IBS is glycomacropeptide (GMP).

**Figure 1:**
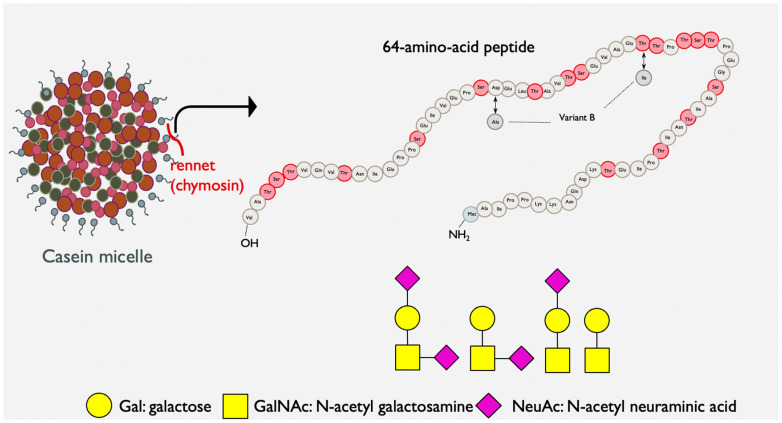
Structural representation of bovine κ-casein glycomacropeptide (GMP) highlighting its amino acid sequence and predominant O-linked glycans. Glycan symbols: yellow square, *N*-acetyl galactosamine; yellow circle, galactose; and purple diamond, *N*-acetyl neuraminic acid.

GMP, a 64-amino acid fragment derived from bovine κ-casein during cheesemaking, constitutes 20–25% of whey protein and exists in various forms with distinct genetic variants and post-translational modifications, including glycosylation (Figure 1) [42,43,44,45]. It undergoes glycosylation with 11 different O-linked glycan structures attached primarily to the threonine and serine residues in the peptide [46]. Commercial extraction from sweet whey involves processes such as ultrafiltration and ion exchange chromatography [47]. Due to its deficiency in certain aromatic amino acids, GMP is a significant dietary source for individuals with phenylketonuria (PKU), a disorder impairing phenylalanine metabolism [48]. Present in dairy products including milk and yogurt, albeit in lower concentrations than isolated sweet whey protein, GMP is also released in the consumer’s gut from κ-casein by gastric pepsin [49,50].

**Figure 2:**
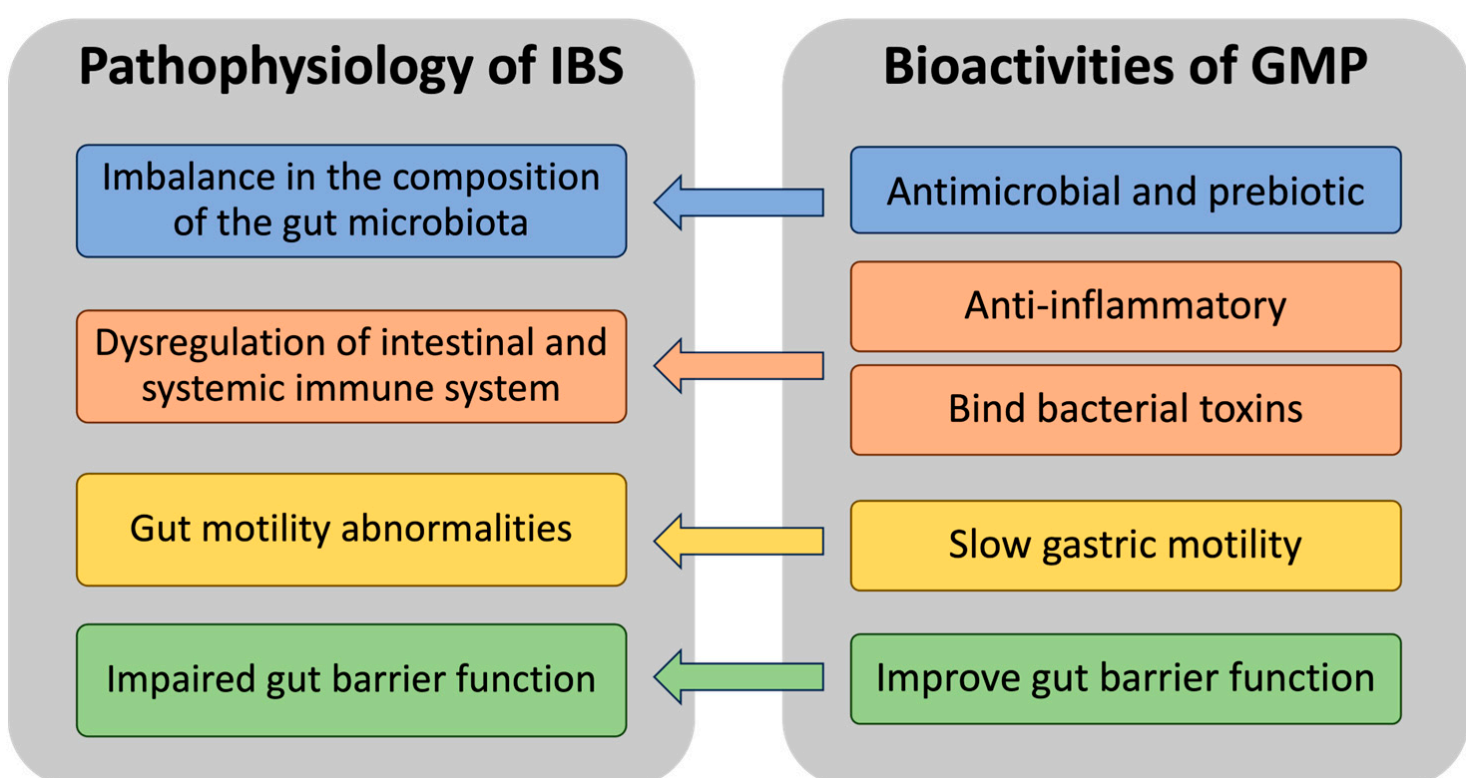
GMP’s diverse bioactivities and their potential relevance in targeting the pathophysiological aspects of IBS.

In vitro and animal studies have attributed several health-promoting bioactivities to GMP, including antimicrobial and prebiotic activities, immunomodulatory properties, and toxin-binding capabilities, highlighting its potential for mitigating gastrointestinal symptoms prevalent in conditions similar to IBS [51,52,53,54,55,56,57]. GMP’s potential role remains largely unexplored in the evolving landscape of IBS treatment research. In this review, we aim to critically evaluate GMP’s influence on the gut microbiome, immune responses, gut motility, barrier functions, and toxin-binding, assessing its potential to address the altered microbiome, immune regulation, and gut function in IBS patients (Figure 2).

## 2. GMP and the Microbiome in IBS

### 2.1. Gut Microbiome in IBS

The gut microbiome plays a crucial role in developing and maintaining human health.

Studies on microbiota diversity in IBS patients have yielded variable results. The microbiomes of those with IBS often exhibit lower diversity than those who are healthy, indicating a reduced number of different bacterial species, a condition referred to as dysbiosis [3,8,36,58,59,60,61,62,63]. However, other studies have found no significant difference in diversity between IBS patients and healthy individuals [64,65,66,67]. These inconsistent findings highlight the complexity of the relationship between microbial diversity and IBS. A better understanding of the factors affecting microbial diversity among individuals with IBS and their implications for its development and management is needed.

Some studies indicate that individuals with IBS have specific family-, genus- or species-level differences in their microbiota compared with healthy individuals, although the specific differences identified have varied between studies. Pittayanon et al. recently reviewed these microbial differences in people with IBS and found a set of common differences across studies, including increased family *Enterobacteriaceae* (belonging to the phylum Proteobacteria), family *Lactobacillaceae* and genus *Bacteroides*, decreased unculturable Clostridiales I, and genus *Faecalibacterium* (including *Faecalibacterium prausnitzi* and genus *Bifidobacterium*) [68].

Changes in the gut bacterial composition of patients with IBS may be partially responsible for their observed symptoms. For example, in patients with an IBS type characterized by constipation (IBS-C), the abundance of methane-producing bacteria *Methanobacteriales* is higher than in healthy individuals [60]. This association may be due to methane’s ability to decrease intestinal peristalsis and increase transit time [69]. Conversely, in patients with IBS-D, the abundance of *Methanobacteriales* is lower than in healthy controls [60,69], which could lower gut methane production and decrease transit time [69]. Furthermore, IBS-D patients were observed to have higher abundances of hydrogen sulfide-producing bacteria [70]. The higher hydrogen sulfide production may be partially responsible for symptoms observed in IBS-D, as previous studies have associated breath hydrogen sulfide levels with diarrhea [71]. Furthermore, rat studies indicate that hydrogen sulfide relaxes smooth muscles [72]. Investigations by Parkes et al. revealed that individuals with IBS exhibit increased rectal mucosa production, which correlates with higher levels of bacteria such as *Bacteroides* and *Clostridia*. These bacteria have a strong affinity for mucin and their proliferation in this environment may have implications for the pathophysiology of IBS [73].

Fecal microbiota transplants have emerged as a valuable tool in this field, providing evidence of the microbial contribution to IBS. El-Salhy et al. conducted a study in which fecal transplants from healthy donors resulted in notable improvements in IBS symptoms and overall quality of life [74]. Similarly, Crouzet et al. demonstrated that transferring stool from IBS-D patients to germ-free rats increased intestinal motility, intestinal permeability, and visceral organ pain sensitivity, all of which are commonly observed in individuals with IBS-D [75].

Some studies have found beneficial effects for managing IBS symptoms from consuming probiotics. For example, studies have indicated the beneficial effects of probiotic strains such as *Bifidobacterium infantis* (*B. infantis*) and *Lactobacillus* in improving symptoms and overall management of IBS [76,77]. A randomized controlled trial conducted by Enck et al. found that a mixture of *Escherichia coli* (*E. coli*) (DSM 17252) and *Enterococcus faecalis* (DSM 16440) led to improved symptoms in people with IBS [78]. Additionally, O’Mahony et al. found that supplementing IBS patients with the probiotic strain *B. infantis* 35624 for eight weeks reduced symptom scores for abdominal pain/discomfort, bloating/distention, and bowel movement difficulty. Compared to the placebo group, it also normalized the abnormal ratio of the anti-inflammatory cytokine IL-10 to the proinflammatory cytokine IL-12 in IBS patients [77]. These results suggest that *B. infantis* 35624 can alleviate symptoms and modulate the immune response in individuals with IBS. However, other studies have shown no improvement [79,80,81]. For example, several trials have investigated the effects of specific *Lactobacillus* strains on IBS symptoms but found no significant benefits. These trials include those examining the impact of *Lactobacillus casei* (*L. casei*) *GG*, *Lactobacillus plantarum* (*L. plantarum*) *DSM 9843*, and *Lactobacillus salivarius* (*L. salivarius*) UCC43 [79,80,81]. Despite initial expectations, these studies did not demonstrate a notable improvement in symptoms, suggesting that probiotics’ efficacy in managing IBS may vary depending on the specific strains used.

In addition to highlighting differences in microbial composition between healthy individuals and IBS patients, considering the intricate interaction between the gut microbiota and the host’s immune system is also important. The gut microbiota plays a critical role in maintaining immunological homeostasis, and alterations in microbial populations associated with IBS may be consequential to immune function. Further exploration of the gut microbiome and its impact on the immune system will be discussed later in this review, providing a comprehensive understanding of the gut microbiome and its potential modulation in managing IBS.

### 2.2. GMP as an Antimicrobial Agent

GMP reportedly exhibits antimicrobial properties, contributing to its potential therapeutic effects (Table 1) [57,82,83,84,85,86]. GMP’s antimicrobial effects do not involve direct killing of the bacteria but rather inhibit the adhesion of bacterial pathogens to intestinal cells [57]. For example, GMP can decrease the attachment of specific strains of enteropathogenic *E. coli* (EPEC) O125:H32, EPEC O111:H2, and enterohemorrhagic *E. coli* (EHEC) 12900 O157:H7 as well as *Salmonella enteritidis* to HT29 and Caco-2 intestinal cell lines [84,85,87]. However, reduced adhesion is highly species-specific, with GMP not inhibiting the adhesion of other *E. coli* strains [87] or *Desulfovibrio desulfuricans* [85], a micro-organism often associated with IBS [88] and inactive inflammatory bowel disease (IBD) [89,90]. Similarly, GMP reduces pathogenic *E. coli* (verotoxigenic *E. coli* and EPEC) strains’ adhesion to human HT29 tissue cell cultures [85]. GMP’s ability to prevent bacterial adhesion is typically attributed to its glycosylation [87]. For example, Nakajima et al. found that GMP’s binding ability to enterohemorrhagic *E. coli* O157:H7 was significantly reduced after removing the sialic acid present in GMP (a process known as desialylation) [84].

Although GMP can be effective in preventing pathogen adhesion to intestinal cells, it can also inhibit the binding of certain probiotic organisms. For example, GMP has been shown to reduce the adhesion of various probiotic Lactobacillus strains, such as *L. pentosus*, *L. casei*, and *L. acidophilus*, but not of *L. gasseri* to HT29 cells [85].

GMP contributes to the enhancement of mucus barrier function by binding to bacteria or mucin proteins directly. The intestinal epithelium is covered with a mucus layer comprising highly glycosylated proteins called mucins, which contribute to gut barrier function [106]. Pathogens such as *Helicobacter pylori* can bind to mucins as a first step in infection [106]. GMP contains multiple sialic acid residues that are similar to glycans on mucins, which are selectively targeted by bacterial lectins. When GMP is present, its glycans compete with the natural mucin glycans in the gut for binding sites on bacterial lectins. This competition can inhibit the adhesion of bacteria to the gut lining. For instance, GMP has been shown to prevent the binding of enterotoxigenic *E. coli K88* fimbriae to mucins [82]. Thus, GMP’s prevention of bacterial adherence can improve barrier function and prevent infection. Supplementing BALB/c mice diets with GMP reduced the abundance of fecal *Enterobacteriaceae* and coliforms [99]. However, there was no significant effect observed on *Enterococcus*. In piglets, oral GMP reduced the percentage of villi with *E. coli* adherence but did not reduce diarrhea [82]. Rong et al. found that supplementing weaning piglets’ diets with 1% GMP mitigated the negative effects of *E. coli* K88 infection, including reduced growth, increased intestinal tissue pathogenic bacteria count, and damage to the intestinal barrier [83]. Additionally, GMP lowered the acute inflammatory response induced by the infection. These findings suggest that GMP has potential beneficial effects on improving gut health and reducing the impact of bacterial infections in piglets. However, GMP’s specific effects on diarrhea symptoms may vary. Further research is needed to fully understand its mechanisms of action and potential applications in human subjects with conditions such as IBS.

### 2.3. GMP as a Prebiotic

In vitro and animal studies indicate that GMP’s prebiotic properties can enhance populations of beneficial bacteria, i.e., *Bifidobacteria* and *Lactobacillus* [53,56,96,99,101] (Table 1).

Studies indicate that GMP’s glycan and peptide moiety may be responsible for its growth-promoting abilities. The glycosylation of GMP is often considered the basis for its prebiotic activity. For example, O’Riordan et al. found that GMP’s bifidogenic effect and transcriptional response were significantly reduced upon removing glycans via periodate oxidation [104]. However, some studies indicate that the peptide moiety has prebiotic effects. For example, Robitaille et al. found that glycosylated, unglycosylated, and mixed GMP were equally effective at fostering the growth of the probiotics *L. rhamnosus* RW-9595-M and *B. thermophilum* RBL67 in culture media compared to the control [53]. GMP appears to retain its prebiotic actions even after partial digestion. For example, Tian et al. found that GMP digested by trypsin had higher growth-promoting effects on BB12 than intact GMP [102]. After a 4-week intervention, Yu et al. found that the formula was enriched with short-chain galacto-oligosaccharides and long-chain fructo-oligosaccharides (scGOS/lcFOS). GMP also promoted an increased abundance of Bifidobacterium in the gut microbiota of preterm infants [93], but the probiotic effect observed may not only derive from the GMP component.

Therefore, GMP may help support beneficial bacteria growth, which can help crowd out pathogens. This function is important because a balanced microbiota is crucial for overall gut health.

### 2.4. GMP’s Influence on the Gut Microbiome

GMP has the potential to ameliorate dysbiosis (Table 1). For example, GMP supplementation increased the microbial diversity in an ex vivo fecal culture model of elderly subjects with lower microbial diversity than the lactose control [103]. Similarly, GMP hydrolysate supplementation helped restore microbial diversity in mice with type 2 diabetes [98]. Specifically, GMP hydrolysate supplementation lowered *Helicobacteraceae* levels; increased *Ruminococcaceae*, the *Bacteroidales_S24-7_group*, *Ruminiclostridium*, *Blautia*, and *Allobaculum* and decreased the *Firmicutes*:*Bacteroidetes* ratio. This finding is relevant to IBS, as patients with IBS had a 1.2–3.5-fold higher ratio of *Firmicutes:Bacteroidetes* compared to healthy controls [38,67,107,108,109]. Sawin et al. demonstrated that providing a GMP-based diet to weaned PKU and wild-type C57BL/6 mice reduced Proteobacteria, specifically the genera *Desulfovibrio*, associated with IBD [110], compared to casein- or amino acid-based diets [95]. Sawin et al. also found that the GMP diet increased the cecal concentrations of SCFAs, including acetate, propionate, and butyrate, in both mouse types compared to the casein and amino acid diets. These increases in SCFA levels may lead to improved intestinal barrier function [111] and reduced systemic inflammation [112]. However, in a murine model “humanized” with human fecal microbiota, GMP derived from bovine milk did not exhibit prebiotic activity on fecal microbiota [97].

GMP’s influence on the human gut microbiome has been explored in multiple contexts, yet the outcomes vary and are often contingent upon the study demographics and specifics of the supplementation. In a study with healthy-term infants randomized to different infant formulas, including those enriched in alpha-lactalbumin with varying GMP levels, no significant shifts in bacterial counts were observed across six months [86]. Hansen et al. showed that obese postmenopausal women’s consumption of GMP supplements reduced *Streptococcus* bacteria when taken twice daily and an overall decrease in microbial diversity when taken thrice daily [94]. However, after replacing dietary proteins with GMP in humans with PKU for six months, Montanari et al. found no changes in overall gut microbiome diversity (albeit, with some increases in a few beneficial species) or short-chain fatty acid levels compared to the baseline [92]. Similarly, Wernlund et al. found no significant changes in fecal microbiota composition or SCFA content when comparing healthy adults before and after GMP supplementation (25 g/day) for four weeks and compared to skim milk-supplemented controls [91]. These results suggest that GMP may not substantially influence humans’ gut microbiome. However, the supplementation duration and the specific supplement and dose used could have affected these findings. Further research is necessary to explore GMP’s impact on the gut microbiome in humans.

### 2.5. Potential Implications of GMP-Induced Microbiota Modulation in IBS

Cell and animal studies suggest that GMP can positively influence gut microbiota composition, indicating its value for IBS patients. Since dysbiosis is a common feature of IBS, GMP’s ability to inhibit the adhesion of pathogenic bacteria and promote beneficial bacteria growth could contribute to restoring a healthy gut microbiota balance in these individuals if taken as a supplement. However, these findings suggest that GMP’s ability to inhibit the binding of certain beneficial *Lactobacillus* strains to intestinal cells may counteract these beneficial effects. Moreover, current studies of GMP supplementation in humans provide scarce evidence that GMP can positively modulate the human microbiome. More research is needed to determine the effects of GMP supplementation on the microbiome of IBS patients. Since GMP is known to be digested partially within the human gut [113,114,115], encapsulation and other delivery strategies may be needed to enhance its effectiveness.

## 3. GMP and Inflammation in IBS

### 3.1. Inflammation in IBS

Although the precise etiology of IBS remains unknown, inflammation may be a contributing factor [32]. In some studies, low-grade inflammation and immune activation were found in IBS patients [116,117,118]. This inflammation may be instigated by infections, alterations in the gut microbiota, or increased intestinal permeability, potentially causing symptoms such as abdominal pain or altered bowel habits [32]. Moreover, the involvement of the brain–gut axis, which affects neuroendocrine pathways and glucocorticoid receptor genes, could foster a pro-inflammatory state, contributing to the manifestation of IBS symptoms [32].

Some studies have identified cytokine differences between IBS patients and healthy controls [54,119,120,121,122]. More specifically, people with IBS often have decreased levels of anti-inflammatory cytokines such as IL-10 compared to healthy individuals [119,120,123,124]. IL-10 normally regulates and dampens inflammation by inhibiting pro-inflammatory cytokine production and promoting regulatory immune cell activity [124]. However, when IL-10 production is suppressed, the inflammatory process may become prolonged or exaggerated, resulting in chronic or recurrent symptoms and the development of certain conditions, such as IBS. However, not all studies found decreased IL-10 in IBS patients; for example, Vara et al. found that IL-10 levels were higher in people with IBS compared to healthy controls [54].

Studies have indicated that compared to healthy individuals, IBS patients have higher levels of pro-inflammatory cytokines in their plasma. In past studies, cytokines that have appeared at higher levels in IBS patients include (IL)-1, IL-3, IL-4, IL-5, IL-6, IL-8, IL-12, IL-13, IL-16, IL-17, IL-18, tumor necrosis factor-alpha (TNF-α), and interferon-γ (IFN-γ) [32,54,122,125,126]. Higher pro-inflammatory cytokine levels may indicate chronic inflammation in people with IBS [116]. These findings suggest that IBS patients have atypical immune regulation, and more research is needed to understand how the immune system is activated in IBS patients.

Fecal calprotectin (FC) is a calcium- and magnesium-binding protein primarily produced in neutrophils. When found in the intestine or feces, it indicates the presence of neutrophil migration to the inflamed intestinal mucosa [127]. FC is a biomarker of intestinal inflammation. Some studies have reported higher FC levels in a subset of IBS patients, even exceeding those observed in individuals with inflammatory bowel disease (IBD) [128]. Choi and Jeong found higher FC levels in children with IBS than in healthy controls, indicating a potential association between FC and IBS in this population [129]. However, some studies did not find significant differences in FC between IBS patients and controls [130,131]. More research is needed to determine the factors contributing to elevated FC levels in some IBS patients and clarify its role in the pathophysiology of the condition. Understanding the relationship between FC, inflammation, and symptom generation in IBS could provide valuable insights into the underlying mechanisms and potential therapeutic strategies for managing this complex disorder.

Severe viral or bacterial infections can cause inflammation of the gastrointestinal tract (acute gastroenteritis) and induce IBS symptoms that persist even after the pathogen is eliminated from the body (post-infectious IBS) [34]. Post-infectious IBS is characterized by increased T-lymphocytes, mast cells, and cytokines, which can alter gastrointestinal functions, increase intestinal permeability, and potentially cause chronic IBS symptoms [129]. A meta-analysis showed that the risk of developing IBS increased six-fold after a gastrointestinal infection and remained elevated for 2–3 years after the initial infection was resolved [132].

### 3.2. GMP as an Anti-Inflammatory Agent

GMP has anti-inflammatory properties in many cell and animal studies (Table 2). However, some studies indicate that GMP has pro-inflammatory effects in cell and animal studies. Human studies with GMP supplementation indicate a more limited capacity to modulate inflammation (Table 2).

Many studies conducted on different cell lines have indicated GMP’s anti-inflammatory properties. For instance, when murine spleen cells and dendritic cells were challenged with inflammatory agents such as LPS, Concanavalin-A, and Phytohemagglutinin, GMP was found to reduce levels of IL-1β, TNF-α, and IL-6 [143]. Macrophage studies, especially regarding inflammation, have often examined TNF-α, IL-1β, and IL-6 levels as indicators. For example, one study indicated that hydrolyzed GMP (GHP) reduced these cytokines in LPS-stimulated macrophages [144]. Similarly, GHP reduced TNF-α, IL-1β, and IL-6 levels in LPS-stimulated RAW264.7 macrophages [145]. The anti-inflammatory response of GMP was further evident in a study of HT29-MTX and Caco-2 cells. Here, GMP reduced LPS-induced inflammation, which may have been partially responsible for the increase in tight junction proteins and improved intestinal barrier function [147].

GMP has demonstrated anti-inflammatory effects across various animal models. In rats subjected to trinitrobenzenesulfonic acid-induced colitis, GMP administration resulted in decreased IL-1 levels [134]. Similarly, in rats with experimental ileitis, GMP’s reduction in inflammatory markers (IL-1β, TNF-α, IL-17, IL-2, and IL-1Ra) was comparable to the therapeutic effects of the standard drug, 5-aminosalicylic acid [135]. A study on rat splenocytes and Wistar rats also supports GMP’s anti-inflammatory capabilities, wherein GMP decreased levels of IFN-γ and TNF-α [136]. Rats in another investigation showed diminished expression of a host of inflammatory cytokines, including IL-1β, IL-17, IL-23, IL-6, TGF-β, and IL-10, when their diet incorporated GMP [137]. A study with PKU (Pah(enu2)) and wild-type (WT) C57BL/6 mice indicated an anti-inflammatory response to GMP, with decreases in IFN-γ, TNF-α, IL-1β, IL-2, and IL-10 [139]. Similarly, C57BL/6 wild-type and Rag−/− mice showed lowered IL-4, IL-5, and IL-13 levels when fed with GMP [140]. In more recent rat studies, GMP’s effect remained consistent: one highlighted a decline in IL-1β levels following GMP supplementation [141], while another documented reduced levels of IL-1β, TNF-α, IL-5, and IL-13 with GMP in the diet [142].

Though most animal and cell studies have revealed GMP’s anti-inflammatory effects, some show pro-inflammatory effects. For example, in THP-1 cells, GMP treatment elevated levels of IL-8 and IL-1β [136]. Furthermore, GMP exposure in LPS-stimulated RAW264.7 macrophages upregulated IL-1α and TNF-α [148]. Similarly, in C57BL/6 mice, GMP supplementation increased levels of IL-6, TNF-α, and IFN-γ [138]. These findings indicate that while GMP has predominantly shown anti-inflammatory effects, there are instances and conditions where it exhibits pro-inflammatory effects [136,138,148].

Human studies on the immunomodulatory activity of GMP have provided varied findings. GMP supplementation decreased endoscopically observed colonic inflammation when added to standard therapy for individuals with ulcerative colitis. However, despite this localized improvement, the subjects’ plasma cytokine levels were not significantly altered [133]. Extending the research to the broader population, a study with 24 healthy adults indicated that a four-week regimen of GMP supplementation did not lead to any marked immunomodulatory effects compared to skim milk [91]. Similarly, in a targeted study on obese postmenopausal women, GMP supplementation did not induce any prominent changes in immune responses [94].

### 3.3. Potential Implications of GMP-Induced Anti-Inflammatory Modulation in IBS

Since most cell and animal studies indicate that GMP has anti-inflammatory properties (suppressing the production of pro-inflammatory cytokines, such as TNF-α and IL-6 [134,135,136,137,139,141,142,143,144,145,147,148] and promoting the production of anti-inflammatory cytokines, such as IL-10 [136,138,140,148]), GMP supplementation may help treat individuals with chronic inflammation, including people with IBS. This modulation of inflammatory mediators may alleviate symptoms. Since inflammation plays a significant role in IBS pathogenesis, addressing inflammation is essential for symptom management.

Further research is needed to identify the mechanisms underlying GMP’s anti-inflammatory effects in IBS. Rigorous clinical trials are necessary to evaluate the efficacy, optimal dosages, and long-term effects of GMP supplementation in IBS patients.

## 4. GMP’s Toxin Binding, Gut Motility-Decreasing, and Barrier Function-Enhancing Properties in IBS

GMP has toxin binding, gut motility-decreasing, and barrier function-enhancing properties (Table 3) that may help manage IBS symptoms.

### 4.1. Binding Toxin

Some gut bacteria (i.e., *Campylobacter*, *Shigella*, *E. coli*, and *Salmonella*) can produce toxins, including endotoxins and exotoxins. These toxins can stimulate the immune system and cause inflammation, disrupting gut motility and permeability. These toxins and their effects may contribute to the symptoms and pathology of post-infectious irritable bowel syndrome (PI-IBS) [152], including visceral hypersensitivity [153].

LPS is one of the most concerning endotoxins produced by certain Gram-negative bacteria (i.e., *Bacteroidales*). High levels of LPS can increase the permeability of the gut barrier [154]. This increased permeability allows LPS and other toxins to enter the bloodstream, causing systemic inflammation. The presence of LPS in the gut may contribute to the development and maintenance of IBS symptoms. Studies have shown that patients with diarrhea-predominant IBS (IBS-D) exhibit significantly higher serum levels of LPS [155]. Binding and neutralizing these toxins could help manage IBS symptoms by decreasing gut permeability and inflammation.

In vitro studies have demonstrated that GMP can bind to bacterial toxins (Table 3), such as LPS [147] and *Vibrio cholerae*-produced cholera toxin (CT) [55]. GMP and host cells compete for the same binding sites on bacterial toxins. When GMP binds to these harmful toxins, it prevents them from interacting with host cells. GMP can inhibit the binding of CT to Chinese hamster ovary (CHO-K1) cells and ganglioside GM1, which serve as the binding site for CT, and reduce CT-induced morphological changes in the cells [55]. Feeding mice 1 mg of GMP per day protected almost all mice from diarrhea caused by CT. These findings emphasize GMP’s potential as a preventive measure against toxin-mediated gastrointestinal symptoms. GMP can also downregulate the LPS-induced pro-inflammatory response and inhibit the protein expression of NF-κB-p65 in LPS-stimulated cells, potentially mitigating the inflammation induced by LPS [147]. Most studies attribute GMP’s toxin-binding capacity to its glycosylation (particularly sialic acid residues) [55].

GMP’s ability to bind bacterial toxins could have potential implications for managing IBS. By preventing the binding and interaction of toxins with host cells, GMP could help reduce the inflammatory response and alleviate symptoms in individuals with IBS. Further research is needed to explore whether GMP can limit toxin binding in vivo in humans and reduce IBS symptoms.

### 4.2. Gut Motility

People with IBS often have dysregulated gut motility, leading to constipation, diarrhea, or a mix of both [156]. Stress, diet, alterations in the gut microbiome, and hormonal changes can influence gut motility [157]. Stress can trigger the release of hormones and neurotransmitters that affect motility [157]. Diet can modulate gut motility, with insoluble fiber-rich foods promoting transit [158], high-fat and processed foods potentially slowing digestion [159] and certain carbohydrates (fermentable oligosaccharides, disaccharides, monosaccharides, and polyols) impacting bloating and faster motility [160]. Imbalances in the gut microbiota promote gut motility [157]. Hormonal fluctuations, such as increased or decreased levels of progesterone or estrogen, can affect muscle contractions in the intestinal walls, leading to stronger or weaker contractions resulting in diarrhea, constipation, or alternating bowel habits [161]. In addition to directly inducing bowel habit changes associated with IBS, these factors’ influence on gut motility can lead to changes in the gut microflora, which contribute to the development of IBS [162].

Regarding IBS management, GMP has shown potential in modulating gastric secretion and stomach motility [149,150,151] (Table 3). Intravenous injections of GMP in dogs inhibited gastric secretion and motility [149,150,151]. Whether GMP consumed as a food or supplement has similar effects is unknown.

GMP’s potential to slow gastric motility may be particularly beneficial for people with IBS-D, as it could help modulate the dysregulated motility experienced by these patients. Further research is needed to elucidate the specific mechanisms by which GMP affects gut motility and whether these effects are transferable to humans.

### 4.3. Barrier Function

The intestinal barrier plays a pivotal role in health, as it prevents harmful pathogens and toxins from entering the bloodstream and facilitates the uptake of essential nutrients. This barrier can be compromised in IBS, particularly within its IBS-D and PI-IBS subtypes, leading to symptomatic manifestations such as abdominal pain and bowel disturbances [163]. Gastrointestinal infections can compromise intestinal barrier function, as some pathogens are adept at altering tight junctions [164]. This compromise can facilitate the migration of bacteria and their products from the intestinal lumen into the bloodstream, igniting immune responses and subsequent inflammation [164]. Such inflammatory responses can further exacerbate intestinal permeability [156].

GMP has improved gut barrier function in cell models and animal studies (Table 3). For example, Arbizu et al. found that in HT29-MTX and Caco-2 cell lines, which were subjected to LPS-induced disruptions, GMP treatment upregulated tight junction proteins (claudin-1, claudin-3, occludin, and zonula occludens-1) [147]. Such proteins are essential for preserving the structural integrity of the intestinal barrier. In the same study, GMP was observed to mitigate the permeability induced by TNF-α in Caco-2/HT29-MTX co-cultured monolayers. This mitigation was comparable to the effects of TGF-β1, a protein known to enhance epithelial barrier function [147]. Additionally, Feeney et al. demonstrated that GMP could significantly reduce the adhesion of pathogenic *E. coli* to HT29 and Caco-2, underlining its potential to bolster gastrointestinal barrier defense against harmful bacteria [70].

GMP’s effects on barrier function have been shown in animal models. For example, supplementing GMP to weaning piglets challenged with an *E. coli* K88 helped prevent infection-induced increases in intestinal barrier permeability [83]. This protection contributed to the overall health of the piglets and reduced the impact of infection.

By enhancing the integrity of the intestinal barrier, GMP can prevent the translocation of harmful substances and pathogens, which could help prevent inflammation and promote overall gut health. These findings suggest that GMP may hold promise for improving intestinal barrier function in individuals with IBS. Further research is needed to determine whether GMP can enhance barrier function in humans and whether this improved barrier function can support IBS management.

## 5. Conclusions and Future Perspective

The current body of research indicates that the diverse properties of GMP (antimicrobial, prebiotic, immunomodulatory, toxin-binding, gut motility-modulating, barrier function-enhancing) align well with the recognized pathophysiological aspects of IBS (microbiome imbalances, immune system alterations, altered gut function). Therefore, GMP may have potential as a therapeutic agent in IBS management.

GMP was shown to prevent the binding of pathogenic bacteria and toxins to models of the human intestine (HT29 and Caco-2 cancer cell lines). These cell lines can differentiate into cells resembling normal intestinal enterocytes, serving as useful models for research. However, the results of these cell line experiments may not accurately reflect human physiology. Future work should also examine the extent to which GMP exerts these effects on primary cell lines and enteroids.

Current research predominantly indicates that GMP has anti-inflammatory activity, which could be useful in alleviating chronic inflammation often associated with IBS. Although some studies indicate its pro-inflammatory effects, further investigations are needed to clarify GMP’s immunomodulatory effects and identify their precise mechanisms.

GMP’s toxin-binding, gut motility-modulating, and barrier function-enhancing properties may be useful in IBS management. Studies have demonstrated GMP’s ability to bind and neutralize harmful bacterial toxins, which could prevent characteristic symptoms of IBS, including toxin-induced inflammation and increased gut permeability. GMP’s ability to decrease gut motility could be useful in cases of IBS-D. GMP’s ability to enhance intestinal barrier function could also help mitigate symptoms associated with IBS.

However, no direct studies have investigated the effects of GMP supplementation on the symptoms, microbiome, immune profile, and gut health of individuals with IBS. Clinical trials into GMP’s effects on these aspects of IBS management are needed. Future research should also examine GMP’s optimal dosage, formulation, and treatment duration for IBS treatment. Moreover, research should examine how GMP’s effects differ across various IBS subtypes, which could facilitate the development of subtype-specific therapeutic approaches. By focusing on these critical areas, research communities can develop a new therapeutic strategy for IBS.

## Figures and Tables

**Table 1:** Studies on the effects of GMP on the microbiome.

Study Type	References	GMP Product	Study Model	Effects on Microbiome ^1^
Clinical Trial	Brück et al., 2006 [86]	α-lactalbumin and GMP-enriched infant formulae	Healthy term infants (*n* = 85)	(n) gut microbiota
	Wernlund et al., 2021 [91]	GMP	Healthy adults (*n* = 25)	(n) gut microbiota
	Montanari et al., 2022 [92]	GMP	People with PKU (*n* = 9)	(+) *Agathobacter* spp.; (+) *Subdoligranulum*;(n) for gut microbiota diversity; (n) Short-chain fatty acids (SCFA)
	Yu et al., 2022 [93]	scGOS/lcFOS (9:1) and GMP	Very preterm infants (*n* = 72)	(+) *Bifidobacterium*
	Hansen et al., 2023 [94]	GMP	Obese postmenopausal women (*n* = 13)	(−) *Streptococcus*; (−) α diversity
Animal Study	Sawin et al., 2015 [95]	GMP	Wild-type and PKU mice—fed GMP	(−) *Proteobacteria*; (−) *Desulfovibrio*; (+) SCFA
	Jiménez et al., 2016 [96]	GMP	Rats—fed	(+) *Lactobacillus*; (+) *Bifidobacterium*; (+) *Bacteroides*
	Ntemiri et al., 2019 [97]	GMP	Mice with humanized fecal microbiota—fed	(n) gut microbiota
	Yuan et al., 2020 [98]	GHP	C57BL/6J mice with induced type 2 diabetes—fed	(+) Diversity of gut microbiota; (−) *Firmicutes:Bacteroidetes* ratio; (+) *Bacteroidales_S24-7*; (+) *Ruminiclostridium*; (+) *Blautia*; (+) *Allobaculum;* (−) *Helicobacteraceae*
	Chen et al., 2012 [99]	GMP	BALB/c mice—fed	(+) *Lactobacillus*; (+) *Bifidobacteria*; (−) *Enterobacteriaceae*; (−) coliforms; (n) *Enterococcus*
	Gustavo Hermes et al., 2013 [82]	GMP	Piglets—fed	(−) *E. coli* attachment to intestinal mucosa; (+) *Lactobacillus*; (−) *Enterobacteria*; (−) villi with *E. coli* adherence
	Rong et al., 2015 [83]	GMP	Piglets—fed	(−) Intestinal barrier permeability damage caused by *E. coli* K88 infection; (−) Acute inflammatory response induced by *E. coli* K88 infection
	Wu et al., 2020 [100]	GMP	Sow and piglet model—fed	(+) *Prevotella*; (+) *Fusobacterium*; (+) *unclassified_f__Prevotellaceae*; (+) *norank_f__Ruminococcaceae*; (+) *Christensenellaceae_R-7_group*; (+) *Ruminococcaceae_UCG-005*; (+) *Ruminococcaceae_UCG-010*
Cell study	Nakajima et al., 2005 [84]	GMP	Caco-2 cells	(−) Adhesion of *Salmonella enteritidis* and enterohemorrhagic *E. coli O157:H7* to Caco-2 cells
	Rhoades et al., 2005 [85]	GMP	HT29 cells	(−) Adhesion of pathogenic *E. coli* (VTEC and EPEC) strains to human HT29 tissue cell cultures; (−) Adhesion of *Lactobacillus pentosus* (*L. pentosus*), *Lactobacillus acidophilus* (*L. acidophilus*), and *L. casei* strains; (n) Adhesion of *Desulfovibrio desulfuricans* or *Lactobacillus gasseri* (*L. gasseri*)
	Brück et al., 2006 [57]	α-lactalbumin and GMP	Caco-2 cells	(−) Adhesion of Enteropathogenic *E. coli* (EPEC), *Salmonella typhimurium* and *Shigella flexneri*
	Feeney et al., 2017 [87]	GMP	HT29 and Caco-2 cells	(−) Epithelial cell barrier dysfunction; (−) pathogen adhesion of Enterohemorrhagic *E. coli (EHEC)* and Enteropathogenic *E. coli (EPEC)*
Culture and medium study	Azuma et al., 1984 [101]	GMP	Bacterial culture of *B. infantis*S12	(+) *B. infantis*S12
	Brück et al., 2003 [56]	GMP and α-lactalbumin	Bacterial culture	(+) *Bifidobacteria*; (+) *Lactobacilli*; (−) *Bacteroides*; (−) *Clostridia*; (−) *E. coli*
	Robitaille et al., 2013 [53]	GMP	Bacterial culture	(+) *Lactobacillus rhamnosus (L. rhamnosus)*; (+) *Bifidobacterium thermophilum (B. thermophilum)*
	Tian et al., 2015 [102]	GHP	Yogurt	(+) *Bifidobacterium animalis* spp. *Lactis* BB12 (BB-12); (+) *Streptococcus thermophilus*; (n) *Lactobacillus bulgaricus*
	Ntemiri et al., 2017 [103]	GMP	Artificial colon model	(+) *Coprococcus*; (+) *Clostridium cluster XIVb*; (+) Fecal microbiota diversity
	O’Riordan et al., 2018 [104]	GMP	Bacterial culture	(+) *Bifidobacterium longum* ssp. *infantis*
	Morozumi et al., 2023 [105]	GMP	GMP containing medium	(+) *Bifidobacterium bifidum*; (+) *Bifidobacterium breve*

^1^ (+) Indicates an observed positive change in the microbiome, such as an increase in abundance or effect; (−) Indicates an observed negative change in the microbiome, such as a decrease in abundance or effect; (n) No significant change. Abbreviations used in the table: GMP: Glycomacropeptide; GHP: Hydrolyzed Glycomacropeptide; PKU: Phenylketonuria; SCFA: Short-chain fatty acids; scGOS/lcFOS: short-chain galactooligosaccharides/long-chain fructooligosaccharides; EPEC: Enteropathogenic *E. coli*; EHEC: Enterohemorrhagic *E. coli*; VTEC: Verotoxigenic *E. coli;* BB-12: *Bifidobacterium animalis* spp. *Lactis* BB12.

**Table 2:** Studies on the effects of GMP on inflammation.

Study Type	References	GMP Product	Study Model	Effects on Inflammation ^1^
Clinical Trial	Hvas et al., 2016 [133]	GMP	People with ulcerative colitis (*n* = 24)	(n) Cytokine levels (−) endoscopic colonic inflammation
	Wernlund et al., 2021 [91]	GMP	Healthy adults (*n* = 24)	(n) No significant change
	Hansen et al., 2023 [94]	GMP	Obese postmenopausal women (*n* = 13)	(n) No significant change
Animal Study	Daddaoua et al., 2005 [134]	GMP	Rats with trinitrobenzenesulfonic acid-induced colitis—fed	(−) IL-1
	Requena et al., 2008 [135]	GMP	Rats with induced ileitis—fed	(−) IL-1β; (−) TNF-α; (−) IL-17; (n) IFN-γ; (−) IL-2; (−) IL-1Ra
	Requena et al., 2010 [136]	GMP	Rat splenocytes and Wistar rats—fed	(+) IL-10; (−) IFN-γ; (−) TNF-α
	López-Posadas et al., 2010 [137]	GMP	Rats—fed	(−) IL-1β; (−) IL-17; (−) IL-23; (−) IL-6; (−) TGF-β; (−) IL-10
	Ortega-González et al., 2014 [138]	GMP	C57BL/6 mice—fed	(+) IL-6; (+) IL-10; (+) TNF-α; (+) IFN-γ
	Sawin et al., 2015 [139]	GMP	PKU (Pah(enu2)) and wild-type (WT) C57Bl/6 mice—fed	(+) Acetate; (+) propionate; (+) butyrate; (−) IFN-γ; (−) TNF-α; (−) IL-1β; (−) IL-2; (−) IL-10
	Muñoz et al., 2017 [140]	GMP	C57BL/6 wild-type and Rag−/− mice—fed	(−) IL-4; (−) IL-5; (−) IL-13; (+) IL-10
	Cervantes-García et al., 2020 [141]	GMP	Rats—fed	(−) IL-1β
	Reyes-Pavón et al., 2020 [142]	GMP	Rats—fed	(−) IL-1β; (−) TNF-α; (−) IL-5; (−) IL-13
Cell study	Mikkelsen et al., 2005 [143]	GMP	Murine spleen cells and dendritic cells challenged with LPS, Concanavalin-A, and PHA	(−) IL-1β; (−) TNF-α; (−) IL-6
	Requena et al., 2010 [136]	GMP	THP-1 cells	(+) IL-8; (+) IL-1β
	Cheng et al., 2015 [144]	GHP	Macrophages	(−) TNF-α; (−) IL-1β; (−) IL-6
	Li et al., 2017 [145]	GHP	LPS-stimulated RAW264.7 macrophages	(−) TNF-α; (−) IL-1β; (−) IL-6
	Foisy-Sauvé et al., 2020 [146]	GMP	Caco-2/15 Cells	(−) Oxidative stress; (−) malondialdehyde; (+) superoxide dismutase 2; (+) glutathione peroxidase
	Arbizu et al., 2020 [147]	GMP	HT29-MTX and Caco-2 cells	(+) Intestinal barrier function; (−) LPS-induced inflammation; (+) Tight junction proteins
	Lu et al., 2022 [148]	GMP	LPS-stimulated RAW264.7 macrophages	(+) IL-1α; (+) TNF-α; (+) IL-10

^1^ (+) Indicates an observed positive change in the microbiome, such as an increase in abundance or effect; (−) Indicates an observed negative change in the microbiome, such as a decrease in abundance or effect; (n) No significant change. Abbreviations used in the table: GMP: Glycomacropeptide; GHP: Hydrolyzed Glycomacropeptide; PHA: Phytohemagglutinin; IL: Interleukin; IL-1Ra: Interleukin-1 receptor antagonist; TNF-α: Tumor Necrosis Factor alpha; IFN-γ: Interferon gamma; TGF-β: Transforming Growth Factor beta; PKU: Phenylketonuria; Pah (enu2): Phenylalanine hydroxylase enzyme mutation in a PKU mouse model; WT: Wild type; LPS: Lipopolysaccharides; Rag−/−: Recombination activating gene knockout mice, which lack mature T and B lymphocytes; THP-1 cells: A human monocyte cell line.

**Table 3:** Studies on the effects of GMP on other functions.

Study Type	References	GMP Product	Study Model	Effects ^1^
Animal Study	Vasilevskaia et al., 1977 [149]	GMP	Dogs—ntravenous injection	(−) Gastric juice secretion
	Stan and Chernikov, 1979 [150]	GMP	Dogs—intravenous injection	(−) Gastric secretion
	Stan et al., 1983 [151]	GMP	Dogs—intravenous injection	(−) Food motility of the stomach fundus; (−) Cyclic-repetitive vomiting; (−) Gastric secretion; (−) Gastric motility
	Rong et al., 2015 [83]	GMP	Piglets—fed	(+) Protection against *E. coli K88*-induced barrier permeability damage
	Wu et al., 2020 [100]	GOS and GMP	Sow and piglet model—fed	(+) Tight junctions and mucins to enhance intestinal barrier functions
Cell study	Kawasaki et al., 1992 [55]	GMP	CHO-K1 cells	(−) Cholera toxin binding; (−) morphological changes
	Arbizu et al., 2020 [147]	GMP	HT29-MTX and Caco-2 cells	(+) Intestinal barrier function; (−) LPS-induced inflammation; (+) Tight junction proteins

^1^ (+) Indicates an observed positive change in the microbiome, such as an increase in abundance or effect; (−) Indicates an observed negative change in the microbiome, such as a decrease in abundance or effect; (n) No significant change. Abbreviations used in the table: GMP: Glycomacropeptide; GOS: Galactooligosaccharides; LPS: Lipopolysaccharides.

## Data Availability

Not applicable.
